# A Flight Direction Design Method for Airborne Spectral Imaging Considering the Anisotropy Reflectance of the Target in Rugged Terrain

**DOI:** 10.3390/s19122715

**Published:** 2019-06-17

**Authors:** Huijie Zhao, Bolun Cui, Guorui Jia

**Affiliations:** School of Instrumentation & Optoelectronic Engineering, Beihang University, Key Laboratory of Precision Opto-Mechatronics Technology, Ministry of Education, 37# Xueyuan Road, Haidian District, Beijing 100191, China; hjzhao@buaa.edu.cn (H.Z.); boluncui@buaa.edu.cn (B.C.)

**Keywords:** hyperspectral imaging, airborne remote sensing, rugged terrain, flight mission planning

## Abstract

An excellent mission plan is the prerequisite for the acquisition of high quality airborne hyperspectral images which are useful for environmental research, mining etc. In order to minimize the radiance non-uniformity caused by the anisotropic reflectance of targets, the flight direction is mostly designed on the solar azimuth or 180° from it for whiskbroom and pushbroom imagers. However, the radiance to the observer is determined not only by the reflectance of the target, but also by the terrain, the illumination direction and the observation direction. So, the flight direction which is defined to minimize radiance non-uniformity might change with the terrain. In order to find the best flight direction for rugged terrain, we firstly analyze the causes of the effect of terrain on radiation non-uniformity based on the radiative transfer process. Then, the flight direction design method is proposed for composite sloping terrain. Tested by digital and physical simulation experiments, the radiance non-uniformity is minimized when the aircraft flies in the designated direction. Finally, a workflow for flight direction planning and optimizing is summarized, considering the flight mission planning techniques and the workflow of remote sensing missions.

## 1. Introduction

Due to the high spectral resolution of hyperspectral images, a large number of applications requiring fine identification of materials or estimation of physical parameters are enabled [1]. As the main equipment for acquiring hyperspectral images, airborne hyperspectral sensors have been widely used in recent decades. Whiskbroom and pushbroom hyperspectral imagers such as AVIRIS [2], HyMap [3], CASI/SASI [4,5], APEX [6] have been adopted for many operational routines. An excellent flight plan is the prerequisite of high imaging quality [7]. Firstly, to guarantee the proper ground sampling distance (GSD), the absolute flight altitude and speed should be calculated with the instantaneous field of view (IFOV) of the imager and the digital elevation model (DEM) of the area of interest (AOI) [8,9]. Secondly, as the AOI cannot be covered with a single flight stripe most of time, several parallel stripes are necessary for the entire flight routine to cover the entire AOI [10]. Then, the proper distance between adjacent stripes is calculated with the field of view (FOV) of imager, the flight height and the DEM of the AOI to guarantee specific overlaps between adjacent stripes [11]. Thirdly, to ensure illumination onto the scene and to limit the presence of shadows, the image is acquired on a sunny day when solar zenith angle is less than 60° [9].

The effect of the bidirectional reflectance of the ground surface is also considered for some missions. Because of the anisotropy in the reflectance of the surface and the difference of observation directions among pixels in different columns, there are radiance gradients in the cross direction of an image and radiance differences in the overlap between adjacent stripes [12,13]. These radiance non-uniformities are more obvious in the data from whiskbroom and pushbroom imagers, as the FOVs of these imagers are often larger than 30°. The radiance gradient in the image and the radiance difference in the overlap between adjacent stripes are often affected by the flight direction. As mentioned by Pepe [10] and Myers [14], the sun glint and the ‘hotspot’ should be avoided when designing the flight direction. The ‘hotspot’ is the heavily reflecting sun light at ‘antisolar point’ [10]. In order to avoid the sun glint on the water, Myers [14] and Montes [15] mentioned that the flight line should be the solar azimuth or 180° from the solar azimuth. Downey [16], Beisl [17], Schiefer [18], Langhans [19], Kötz [20] and Zou [21] also designed the flight direction aligned with the sun-target-observer plane (also called principal plane) to minimize the radiance gradient in the image. Schiefer planned a flight direction which was aligned with the principal plane to get the reference image for the validation of the correction of the bidirectional reflectance–distribution function (BRDF) effects [18].

The radiance gradient and radiance difference reach a minimum while the flight direction is parallel to the principle plane only for the horizontal surface and the azimuthal symmetry BRDF condition. However, as the ground surface is not always horizontal, the reflected radiance also depends on the illumination direction, the observation direction, the facing direction of the surface and the bidirectional reflectance of the surface [22]. For rugged terrain, designing a flight line which is aligned with the principal plane might not be the best choice. In the meantime, as the AOI covered by many stripes, the illumination direction of the sun changes when the imaging process occurs [23]. So, the flight line cannot align with the principle plane all the time. Hence, the design method of flight direction still requires improvements.

In this paper, we analyze the at-sensor radiance from a rugged terrain to find the relationship between the effect of directional reflectance of the surface and the flight direction. Then, a method of flight direction design is developed based on this relationship. To test the performance of the method, two digital simulation experiments over a solo slope scene and a composite slope scene are conducted, respectively, for different illumination directions and observation directions. The physical simulation experiment over the composite slope scene is also conducted with more real targets. Finally, a basic workflow of flight direction design for planning flight mission is developed.

## 2. Methods

**Figure 1:**
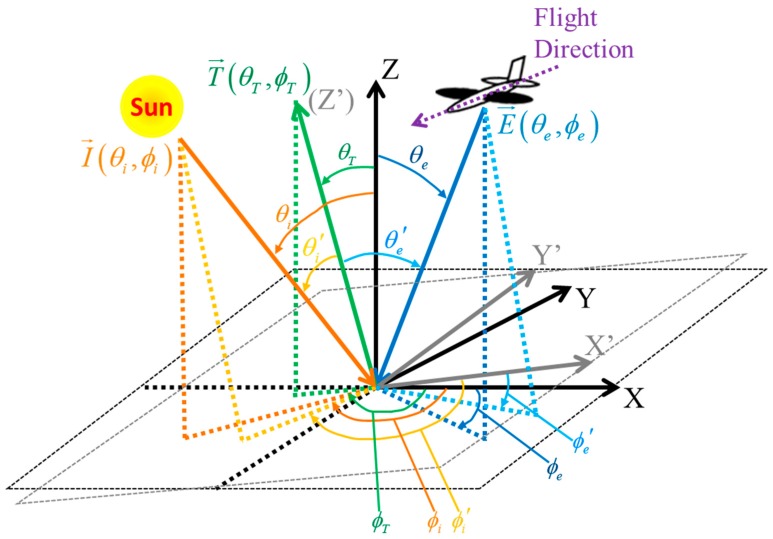
The geometric model for illumination and observation [24].

The radiance gradient in the image and the radiance difference between the overlap areas of adjacent stripes caused by the directional surface reflectance on the rugged terrain is firstly analyzed with radiance transfer process modeling. The illumination-observation geometry is shown in Figure 1. A horizontal Cartesian coordinate system was defined with the target as the origin, *X*-axis pointing to the north and *Z*-axis perpendicular to the earth. For a solo slope as the grey plane in Figure 1, the vector normal to terrain plane is defined as $\vec{T}\left(\theta_{T},\phi_{T}\right)$, where $\theta_{T}$ and $\phi_{T}$ are the slope and aspect of the terrain, respectively. The *Z*’-axis in the local slope coordinate system *X*’-*Y*’-*Z*’ is defined to be aligned with $\vec{T}\left(\theta_{T},\phi_{T}\right)$. The *X*’-axis and *Y*’-axis are rotated from *X*-axis and *Y*-axis according to $\theta_{T}$ and $\phi_{T}$. The illumination direction, observation direction and the relevant angles in these two coordinate systems are listed in Table 1.

The incident vector and exit vector in local slope coordinate system are acquired by rotating the coordinate system by $\theta_{T}$ around *Y*-axis. Then, the new coordinate system is rotated by $-\phi_{T}$ around the new *Z*-axis, as shown in Equations (1) and (2). $R_{Z}\left(\phi_{T}\right)$ and $R_{Y}\left(\theta_{T}\right)$ are the rotation matrixes shown as Equations (3) and (4).

(1) $$\vec{I^{\prime }}\left(\theta_{i}{}^{\prime },\phi_{i}{}^{\prime }\right)=R_{Z}\left(\phi_{T}\right)R_{Y}\left(\theta_{T}\right)\vec{I}\left(\theta_{i},\phi_{i}\right),$$

(2) $$\vec{E^{\prime }}\left(\theta_{e}{}^{\prime },\phi_{e}{}^{\prime }\right)=R_{Z}\left(\phi_{T}\right)R_{Y}\left(\theta_{T}\right)\vec{E}\left(\theta_{e},\phi_{e}\right),$$

(3) $$R_{Z}\left(\phi_{T}\right)=\left[\begin{array}{ccc} \cos\phi_{T} & \sin\phi_{T} & 0\\ -\sin\phi_{T} & \cos\phi_{T} & 0\\ 0 & 0 & 1 \end{array}\right],$$

(4) $$R_{Y}\left(\theta_{T}\right)=\left[\begin{array}{ccc} \cos\theta_{T} & 0 & -\sin\theta_{T}\\ 0 & 1 & 0\\ \sin\theta_{T} & 0 & \cos\theta_{T} \end{array}\right],$$

Then the angles defining the incident direction and exit direction are shown as Equations (5) to (8).

(5) $$\theta_{i}{}^{\prime }=\arccos\left(\sin\theta_{T}\cos\phi_{T}\sin\theta_{i}\cos\phi_{i}+\sin\theta_{T}\sin\phi_{T}\sin\theta_{i}\sin\phi_{i}+\cos\theta_{T}\cos\theta_{i}\right),$$

(6) $$\theta_{e}{}^{\prime }=\arccos\left(\sin\theta_{T}\cos\phi_{T}\sin\theta_{e}\cos\phi_{e}+\sin\theta_{T}\sin\phi_{T}\sin\theta_{e}\sin\phi_{e}+\cos\theta_{T}\cos\theta_{e}\right),$$

(7) $$\phi_{i}{}^{\prime }=\arctan\frac{-\sin\theta_{i}\sin\left(\phi_{T}-\phi_{i}\right)}{\cos\theta_{T}\sin\theta_{i}\cos\left(\phi_{T}-\phi_{i}\right)-\sin\theta_{T}\cos\theta_{i}},$$

(8) $$\phi_{e}{}^{\prime }=\arctan\frac{-\sin\theta_{e}\sin\left(\phi_{T}-\phi_{e}\right)}{\cos\theta_{T}\sin\theta_{e}\cos\left(\phi_{T}-\phi_{e}\right)-\sin\theta_{T}\cos\theta_{e}},$$

**Figure 2:**
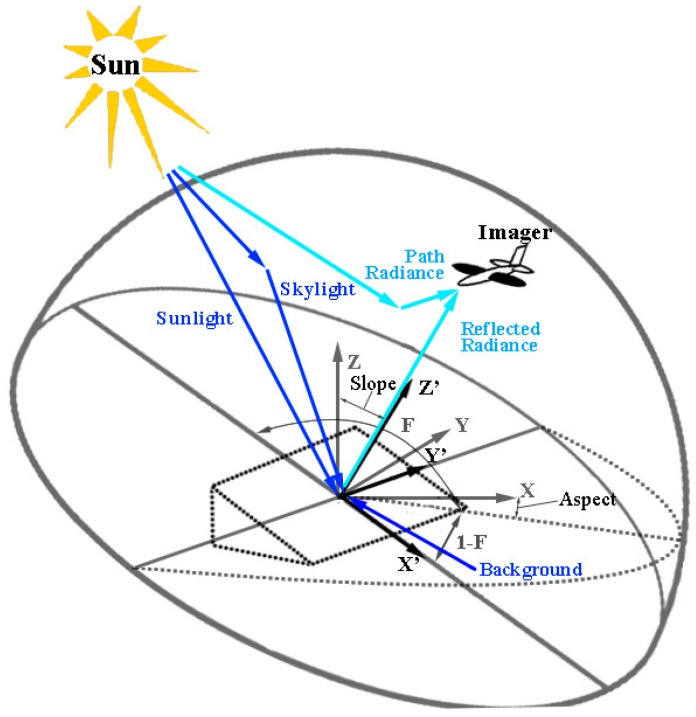
The radiative transfer process [24].

The radiative transfer process is shown in Figure 2. For rugged terrain, the target is illuminated by the direct solar light (sunlight), downward scattered light (skylight) and background reflected light which are the three adding items in the bracket on the right side of Equation (9) [24]. Then the radiance reflected by the target transfers to the observer through the atmosphere, combining with the upwelled scattered radiance (path radiance), as shown in Equation (9) [24]. ${E^{\prime }}_{s\lambda }$ represents the exoatmospheric spectral solar irradiance coming from $\theta_{i}{}^{\prime },\phi_{i}{}^{\prime }$. $L_{d\lambda }$ denotes the skylight radiance to the target from the direction σ,ϕ. $L_{b\lambda }$ denotes the radiance reflected from the background toward the target. F and 1-F are the shape factors denoting the fraction of the hemisphere above the target, which are sky and background respectively, as shown in Figure 2 [24]. $L_{u\lambda }$ denotes the path radiance toward the observer. $\tau_{1}$ and $\tau_{2}$ denote the transmittance from top of atmosphere (TOA) to the target and from target to the sensor. $\rho \left(\theta_{i}{}^{\prime },\theta_{e}{}^{\prime },\phi^{\prime },\lambda \right)$ is the bidirectional reflectance factor (BRF) of the target in the local slope coordinate system.

(9) $$L_{\lambda }\left(\theta_{e},\phi_{e}\right)=\tau_{2}\left(\lambda \right)\left(\begin{array}{l} {E^{\prime }}_{s\lambda }\cos\theta_{i}{}^{\prime }\tau_{1}\left(\lambda \right)\frac{\rho \left(\theta_{i}{}^{\prime },\theta_{e}{}^{\prime },\phi^{\prime },\lambda \right)}{\pi }+\int_{F}L_{d\lambda }\left(\sigma ,\varphi \right)\cos\sigma \frac{\rho \left(\sigma ,\theta_{e}{}^{\prime },\varphi ,\lambda \right)}{\pi }d\Omega \\ +\int_{1-F}L_{b\lambda }\left(\sigma ,\varphi \right)\cos\sigma \frac{\rho \left(\sigma ,\theta_{e}{}^{\prime },\varphi ,\lambda \right)}{\pi }d\Omega \end{array}\right)+L_{u\lambda },$$

(10) $$\phi^{\prime }=\phi_{e}{}^{\prime }-\phi_{i}{}^{\prime },$$

The Hapke model is often adopted to simulate the BRDF of rock or desert [25]; as for vegetation, the SAIL model is often used [26]. The BRF of a surface can be expressed as its corresponding BRDF times π shown as Equation (11) [27]. Even though these models can simulate the BRF of targets precisely, many parameters are needed for a correct simulation. The fill factor, the phase function and the scattering albedo of sand particles are needed for Hapke model [28]. The leaf area, leaf inclination distribution, reflectance and transmittance of leaf, soil reflectance and illumination condition are needed for SAIL model [29]. However, these parameters are not always available. So, a semi-empirical kernel model for rock and vegetation is chosen to calculate the BRF of target [30], as shown in Equation (12). The volume kernel $K_{vol}\left(\theta_{i},\theta_{v},\phi \right)$ and the geometric kernel $K_{geo}\left(\theta_{i},\theta_{v},\phi \right)$ are functions of illumination direction and observation direction. $f_{vol}\left(\lambda \right)$ and $f_{geo}\left(\lambda \right)$ are the factors of kernels. $\rho_{iso}\left(\lambda \right)$ is the isotropic reflectance of the target. The RossThick and LiSparse kernels are used, as shown in Equation (13). The phase angle ζ is the angle between illumination direction and observation direction. t is a variable depending on particle shapes and the direction $\left(\theta_{i}{}^{\prime },\theta_{e}{}^{\prime },\phi^{\prime }\right)$ [30].

(11) $$r_{BRDF}=\frac{\rho }{\pi \left(sr\right)},$$

(12) $$\rho_{BRF}\left(\theta_{i}{}^{\prime },\theta_{e}{}^{\prime },\phi^{\prime },\lambda \right)=\rho_{iso}\left(\lambda \right)+f_{vol}\left(\lambda \right)K_{vol}\left(\theta_{i}{}^{\prime },\theta_{e}{}^{\prime },\phi^{\prime }\right)+f_{geo}\left(\lambda \right)K_{geo}\left(\theta_{i}{}^{\prime },\theta_{e}{}^{\prime },\phi^{\prime }\right),$$

(13) $$\left\{ \begin{array}{l} K_{vol}=\frac{4}{3\pi }\frac{1}{cos\theta_{i}{}^{\prime }+cos\theta_{e}{}^{\prime }}\left[\left(\frac{\pi }{2}-\zeta \right)cos\zeta +sin\zeta \right]-\frac{1}{3}\\ K_{geo}=\frac{1}{\pi }(t-sin\;t\;cos\;t)\left(\frac{1}{cos\theta_{i}{}^{\prime }}+\frac{1}{cos\theta_{e}{}^{\prime }}\right)-\left(\frac{1}{cos\theta_{i}{}^{\prime }}+\frac{1}{cos\theta_{e}{}^{\prime }}\right)+\frac{1+cos\zeta }{2cos\theta_{i}{}^{\prime }cos\theta_{e}{}^{\prime }} \end{array}\right.,$$

**Figure 3:**
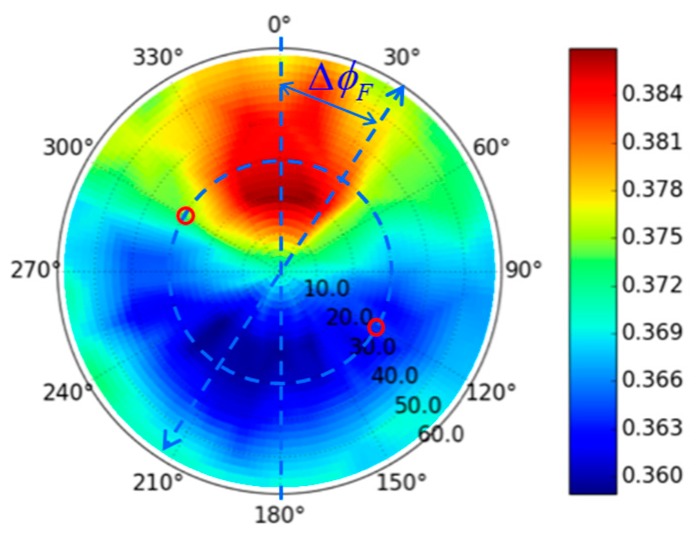
The BRF of sand* [31]. *The azimuth angle changing on circumferential direction align with the blue circle; the zenith angle changing on the radius direction; the blue arrow denotes the flight direction; for specific flight direction, the view directions of two stripes on the same target are the red circles.

According to the radiance transfer process, the radiance reaching the observer for each pixel can be simulated. The radiance gradient in the image and the radiance difference in the overlap area between adjacent stripes can be defined based on the radiance at nadir $L_{\lambda }\left(0,\phi_{e},\lambda \right)$. Assuming the nadir direction of observer is normal to the earth, the maximum radiance difference in an image would appear at the edge of the image, with a view zenith angle of $\pm \frac{FOV}{2}$. For the flight direction $\phi_{F}$ shown as Equation (14), the radiance gradient $\Delta L_{\lambda -\mathrm{intra}}\left(\phi_{F},\lambda \right)$ is defined with the greatest radiance difference in the image as shown in Equation (15). As to the overlapping area, the observation zenith angles of adjacent stripes change slightly for different pixels. The angle between two observation directions comes to the maximum when the observation zenith angles are equal. For the extreme case, the radiance difference between the overlap area of two stripes $\Delta L_{\lambda -\mathrm{inter}}\left(\phi_{F},\lambda \right)$ is defined for the condition that only the edge pixel overlaps, as shown in Equation (16). The BRF of sand is shown as Figure 3 [31]. In order to analyze the relationship between radiance difference caused by the BRF of target and the flight direction in the local slope coordinate system, the observation directions are firstly labeled in Figure 3 for specific flight direction. The flight direction is labeled as the blue arrow with a relative flight direction $\Delta \phi_{F}$ defined as Equation (17). $\phi_{F}{}^{\prime }$ is the flight direction in the local slope coordinate system. Refering to Equation (2), $\phi_{F}{}^{\prime }$ is calculated as Equation (18) by transforming the flight direction vector in horizontal coordinate system to the vector in slope local slope coordinate system. Then, the observation directions are red circles in Figure 3, c.f. Equation (14). It is obvious that the $\Delta L_{\lambda -\mathrm{intra}}\left(\phi_{F},\lambda \right)$ and $\Delta L_{\lambda -\mathrm{inter}}\left(\phi_{F},\lambda \right)$ come to minimum when $\Delta \phi_{F}$ is 0° or 180°.

(14) $$\phi_{F}=\phi_{e}+90^{\circ },$$

(15) $$\Delta L_{\lambda -\mathrm{intra}}\left(\phi_{F},\lambda \right)=\frac{\max\left(\left|L_{\lambda }\left(\frac{FOV}{2},\phi_{e},\lambda \right)-L_{\lambda }\left(0,\phi_{e},\lambda \right)\right|,\left|L_{\lambda }\left(-\frac{FOV}{2},\phi_{e},\lambda \right)-L_{\lambda }\left(0,\phi_{e},\lambda \right)\right|\right)}{L_{\lambda }\left(0,\phi_{e},\lambda \right)},$$

(16) $$\Delta L_{\lambda -\mathrm{inter}}\left(\phi_{F},\lambda \right)=\frac{L_{\lambda }\left(\frac{FOV}{2},\phi_{e},\lambda \right)-L_{\lambda }\left(-\frac{FOV}{2},\phi_{e},\lambda \right)}{L_{\lambda }\left(0,\phi_{e},\lambda \right)},$$

(17) $$\Delta \phi_{F}=\phi_{F}{}^{\prime }-\phi_{i}{}^{\prime },$$

(18) $$\phi_{F}{}^{\prime }=\arctan\frac{\tan\left(\phi_{T}-\phi_{F}\right)}{\cos\theta_{T}},$$

The best flight direction is defined as the $\phi_{F}$ when $\Delta L_{\lambda -\mathrm{intra}}\left(\phi_{F},\lambda \right)$ and $\Delta L_{\lambda -\mathrm{inter}}\left(\phi_{F},\lambda \right)$ reaches a minimum. For horizontal surface, the *X*-*Y*-*Z* coordinate system is the same with the *X*’-*Y*’-*Z*’ coordinate system. Then $\Delta L_{\lambda -\mathrm{intra}}\left(\phi_{F},\lambda \right)$ and $\Delta L_{\lambda -\mathrm{inter}}\left(\phi_{F},\lambda \right)$ come to minimum when the flight direction is parallel to the principle plane. This is consistent with the previous studies [15,16,17,18,19,20]. When the surface is not horizontal, the *X*-*Y*-*Z* coordinate system doesn’t coincide with the *X*’-*Y*’-*Z*’ coordinate system. Then $\Delta \phi_{F}$ changes with slope and aspect of the surface as well as the illumination direction. The best flight direction for different condition is calculated as Equation (19).

(19) $$\phi_{F}=\phi_{T}+\arctan\frac{\cos\theta_{T}\sin\theta_{i}\sin\left(\phi_{T}-\phi_{i}\right)}{\sin\theta_{T}\cos\theta_{i}-\cos\theta_{T}\sin\theta_{i}\cos\left(\phi_{T}-\phi_{i}\right)},$$

For more common conditions, there are several slopes in the AOI, whose slope and aspect angles are different from one another. Then, the best flight direction for each slope is not the same. In order to minimize the $\Delta L_{\lambda -\mathrm{intra}}\left(\phi_{F},\lambda \right)$ and $\Delta L_{\lambda -\mathrm{inter}}\left(\phi_{F},\lambda \right)$ for most pixels, the best flight direction for each pixel in AOI is firstly calculated, and then the probability density function (PDF) $P\left(\phi_{F}\right)$ is estimated with kernel density method [32]. At last, the best flight direction of AOI is defined as the expectation of the best flight direction for each pixel, shown as Equation (20).

(20) $$E\left(\phi_{F}\right)=\int_{\phi_{F}}\phi_{F}\cdot P\left(\phi_{F}\right)d\phi_{F},$$

## 3. The Solo Slope Digital Simulation Experiment

The relationship between the radiance gradient or the radiance difference in the overlap area with the slope of the surface was firstly analyzed with the solo slope digital simulation experiment. According to Equation (8), the radiance received by the sensor was simulated for different slopes and aspects of the surface, the illumination directions and observation directions. $\Delta L_{\lambda -\mathrm{intra}}\left(\phi_{F},\lambda \right)$ and $\Delta L_{\lambda -\mathrm{inter}}\left(\phi_{F},\lambda \right)$ were calculated with Equations (15) and (16), respectively. The sunlight irradiance onto the surface ${E^{\prime }}_{s\lambda }\cos\theta_{i}\tau_{1}\left(\lambda \right)$ and the skylight radiance onto the surface $L_{d\lambda }\left(\sigma ,\varphi \right)$ were calculated with MODTRAN 4.0 code [33]. Mid-Latitude Summer (MLS) and Rural were selected as the atmosphere model and aerosol model respectively. The other simulation parameters are listed in Table 2. For simplicity, the radiance reflected by background was ignored as the terrain doesn’t change dramatically when the slope is less than the solar zenith angle. The target simulated is the gravel in the desert whose diameter is several millimeters. To simulate the reflectance from different illumination directions and observation directions, the kernel factors in Equation (11) were regressed with the BRF collected in a similar desert by using an ASD Fieldspec Pro spectroradiometer [34] between zenith of −40° and +40° with 5° intervals in the principle plane and the cross-principle plane. Considering the commonly-used hyperspectral imager equipped on the airborne [1,2,3,4,5], the observation zenith was set to be ±30° for the edge of FOV and 0° for the nadir as equal to the FOV of HyMap imaging spectroradiometer [3].

**Figure 4:**
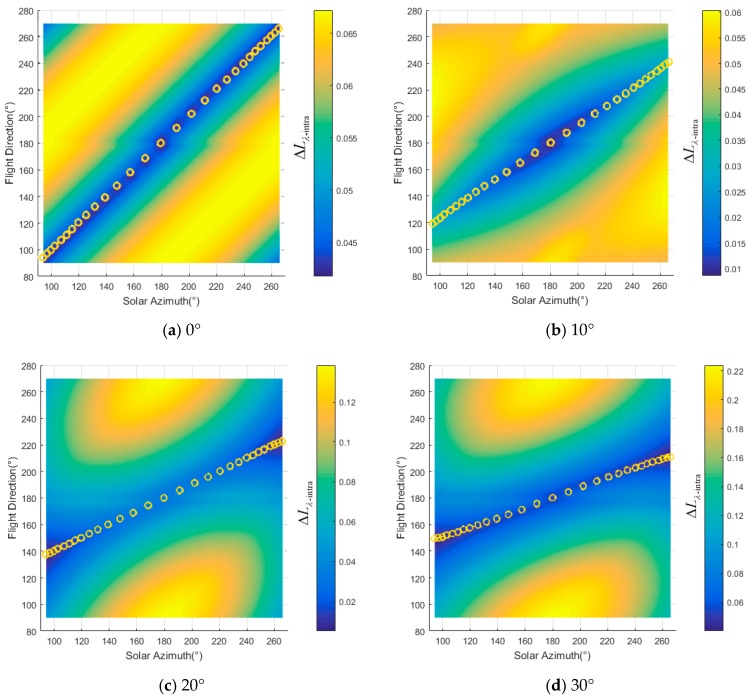

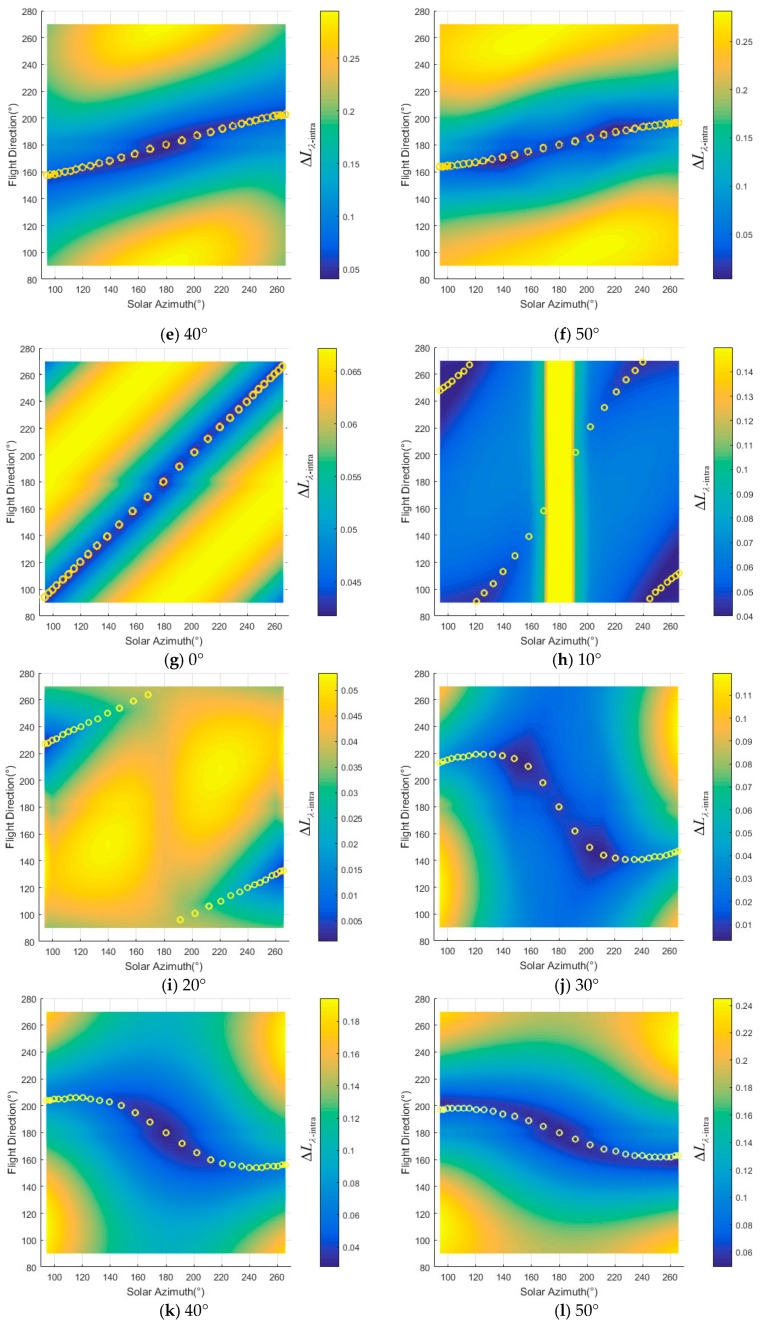
Radiance gradient in the image under different flight direction and solar azimuth: (**a**)–(**f**) the radiance gradient when aspect is 0° and slopes are 0° to 50° respectively, (**g**)–(**l**) the radiance gradient when aspect is 180°and slopes are 0° to 50° respectively.

For different slopes, $\Delta L_{\lambda -\mathrm{intra}}\left(\phi_{F},\lambda \right)$ changes with solar azimuth and flight direction, as shown in Figure 4. The changing of $\Delta L_{\lambda -\mathrm{inter}}\left(\phi_{F},\lambda \right)$ is similar to that of $\Delta L_{\lambda -\mathrm{intra}}\left(\phi_{F},\lambda \right)$. $\Delta L_{\lambda -\mathrm{intra}}\left(\phi_{F},\lambda \right)$ rises with the increase in slope in general. For horizon surface whose $\theta_{T}=0{}^{\circ}$, $\Delta L_{\lambda -\mathrm{intra}}\left(\phi_{F},\lambda \right)$ comes to a minimum when $\phi_{F}=\phi_{i}$ as shown by the yellow circles in Figure 4a,g. This phenomenon coincides with previous studies [15,16,17,18,19,20]. As $\theta_{i}{}^{\prime }$, $\theta_{e}{}^{\prime }$, $\phi^{\prime }$ changing with slope of the surface, the distribution of $\Delta L_{\lambda -\mathrm{intra}}\left(\phi_{F},\lambda \right)$ for different $\phi_{F}$ and $\phi_{i}$ changes significantly. These phenomena show that the flight direction may not be the solar azimuth when surface is not horizontal. For example, as shown in Figure 4l, $\Delta L_{\lambda -\mathrm{intra}}\left(\phi_{F},\lambda \right)$ is 23% when the flight direction is 90° (or 270°) and solar azimuth is 94° (or 266°). The best flight direction should be 200° and 160° when the solar azimuth angles are 94° and 266° respectively and the $\Delta L_{\lambda -\mathrm{intra}}\left(\phi_{F},\lambda \right)$ would be less than 0.06. Calculated with Equation (19), the best fight directions for different azimuth are shown as the yellow dash lines and circles in Figure 4.

**Figure 5:**
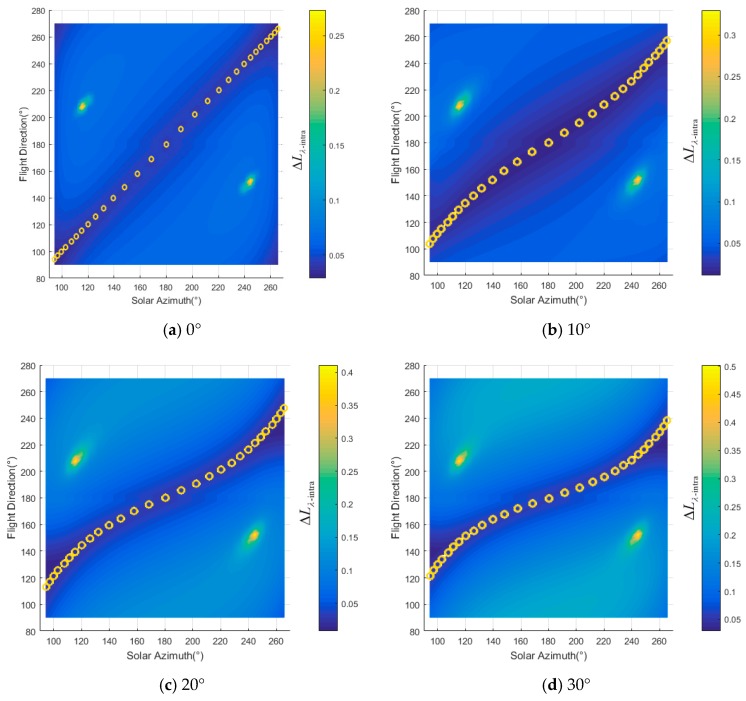

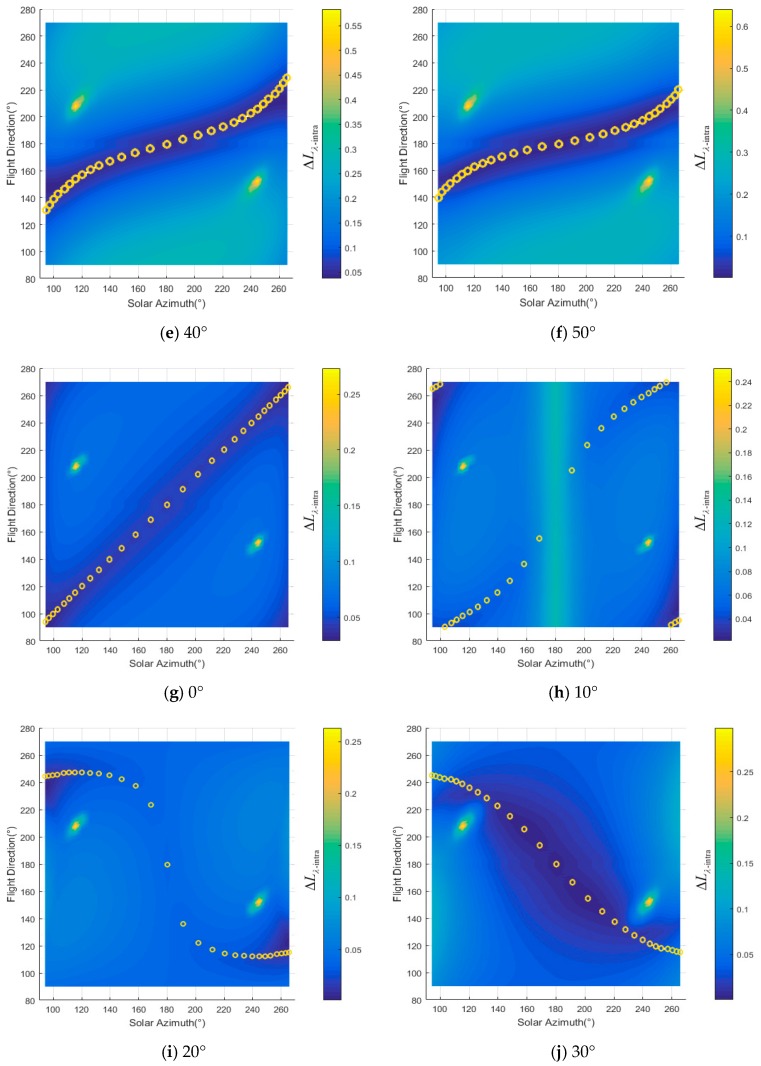

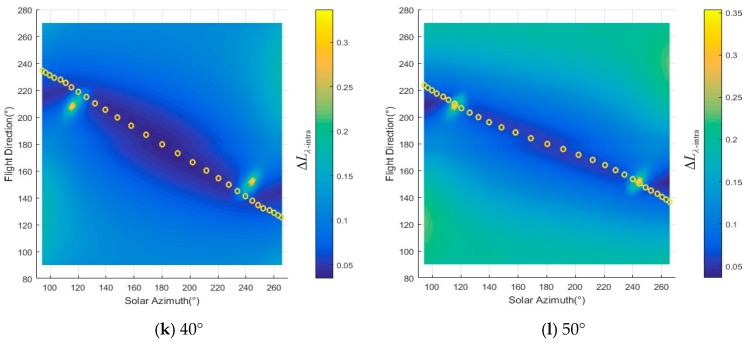
Radiance gradient for different illumination directions and flight directions: (**a**)–(**f**) the radiance gradient when aspect is 0° and slopes are 0° to 50° respectively, (**g**)–(**l**) the radiance gradient when aspect is 180°and slopes are 0° to 50° respectively.

The above experiment didn’t consider the changing of the solar zenith angle. The range of the solar zenith angle is affected by target location. The Huangshan copper mine in Xinjiang, China was chosen as the test area [35]. Huangshan copper mine is a typical many porphyry copper–gold mineralization subzone where there are many typical minerals. The latitude of Huangshan copper mine is 40°, which leads to the solar zenith angle changing between 17.4° and 46.9° from summer morning to afternoon. In the meantime, the slope in this scene changes from 0° to 36° with an average of 23°. Under this condition, the effect of shadow is avoided while the terrain is obviously rugged. The solar azimuth is listed in Table 2. $\Delta L_{\lambda -\mathrm{intra}}\left(\phi_{F},\lambda \right)$ and $\Delta L_{\lambda -\mathrm{inter}}\left(\phi_{F},\lambda \right)$ was calculated with the radiance simulated according to Equation (8). The $\Delta L_{\lambda -\mathrm{intra}}\left(\phi_{F},\lambda \right)$ for different solar azimuth and flight direction is shown in Figure 5. The best flight directions were calculated with Equation (19) and are shown as the yellow circles in Figure 5. It is obvious that $\Delta L_{\lambda -\mathrm{intra}}\left(\phi_{F},\lambda \right)$ comes to a minimum on the best directions. Therefore, for solo slope, the best flight directions can be designed with Equation (19).

## 4. The Composite Slope Digital Simulation Experiment

**Figure 6:**
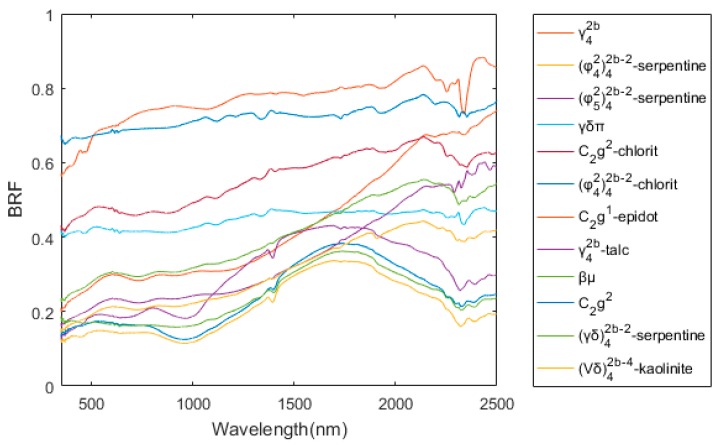
The BRF of samples collected with illumination zenith 45° and view zenith 0°.

**Figure 7:**
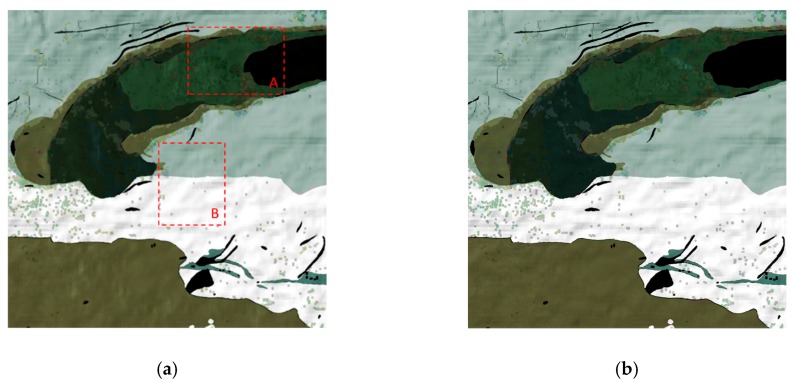
The simulated radiance of the overlap area, (**a**) the image acquired from 60° flight direction and −30° zenith; (**b**) the image acquired on 60° flight direction and +30° zenith.

For more common conditions, an AOI is composed of many slopes; hence, there could be more than one slope in the image. The experiment was conducted to simulate the reflected radiance of the Huangshan copper mine for different illumination directions and observation directions. As the airborne hyperspectral image is affected by terrain and the anisotropic reflectance of surface, the reflectance retrieved from an airborne hyperspectral image contains complex non-uniformity. So, the simulation scene was built with the BRF of simulated rock samples shown in Figure 6 according to the classification map [36], instead of simply using the airborne image. The classification map was developed according to the lithological and mineral distribution in the area [37]. The simulated rock samples were made by mixing mineral powders. And the proportion of mineral powders was similar to the component of rock samples collected in the field. The BRFs of the samples were measured with an ASD FieldSpec Pro spectroradiometer in the laboratory. The DEM of the experiment scene was described by ASTER GDEM data of the AOI. The sunlight irradiance and skylight radiance were calculated with MODTRAN 4.0 model with the parameters listed in Table 3. The radiance reflected by background was ignored. The solar zenith and azimuth angles were set to be consistent with those used in solo slope experiment. The at-sensor radiance images were simulated with Equation (8), as shown in Figure 7.

**Figure 8:**
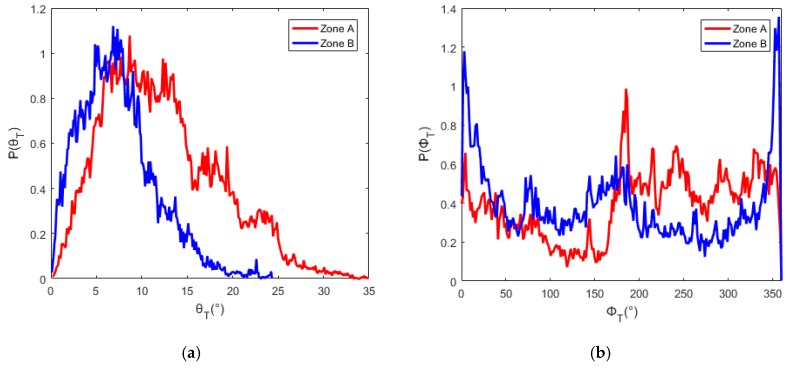
The PDFs of slope and aspect in zone A and B: (**a**) the PDFs of slope, (**b**) the PDFs of aspect.

**Figure 9:**
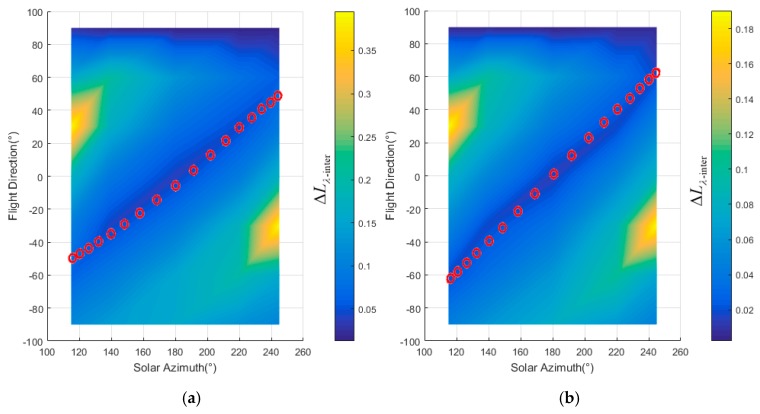
Radiance difference in the overlap area between adjacent stripes: (**a**) the radiance difference in zone A (**b**) the radiance difference in zone B.

Two areas marked as zone ‘A’ and ‘B’ respectively in Figure 7a were defined to test the proposed method of flight direction design. The PDFs of slope and aspect in zones A and B are quite different, as shown in Figure 8. The average slope of zone A is greater than that of zone B (12.5° for zone A and 6.9° for zone B). The slopes in zone A are generally facing west. And most of the slopes in zone B are facing north. $\Delta L_{\lambda -\mathrm{inter}}\left(\phi_{F},\lambda \right)$ for different solar azimuths and flight directions were calculated with Equation (16), as shown in Figure 9. The best flight direction calculated with Equation (20) is shown as the red dash line in Figure 8. Comparing with the distribution of $\Delta L_{\lambda -\mathrm{inter}}\left(\phi_{F},\lambda \right)$, the best flight direction is very close to the direction where $\Delta L_{\lambda -\mathrm{inter}}\left(\phi_{F},\lambda \right)$ coming to the minimum.

## 5. The Composite Slope Physical Simulation Experiment

**Figure 10:**
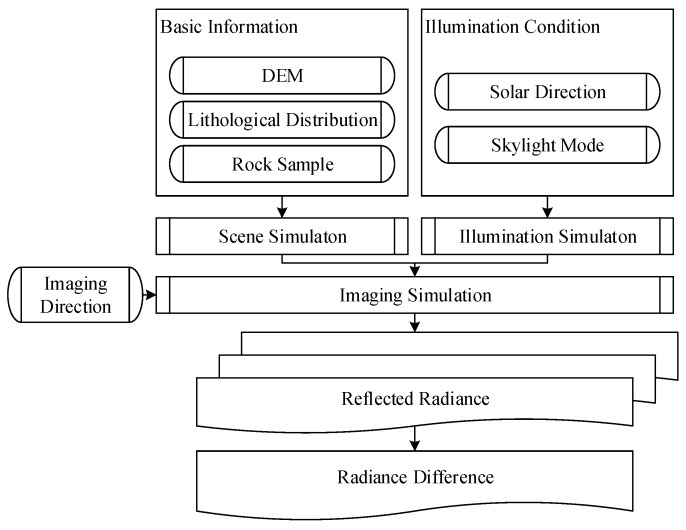
Flowchart of physical simulation experiment.

**Figure 11:**
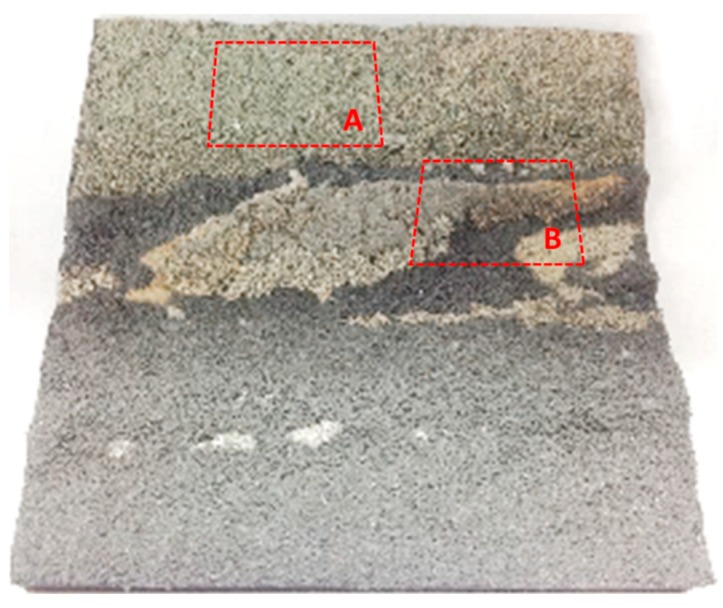
Photo of the physically simulated scene.

**Figure 12:**
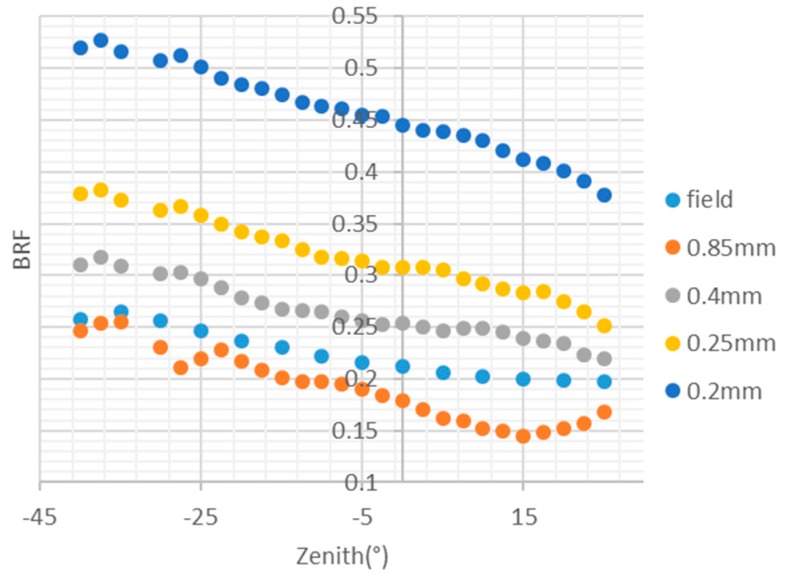
BRF of smashed rock samples of different diameters.

**Figure 13:**
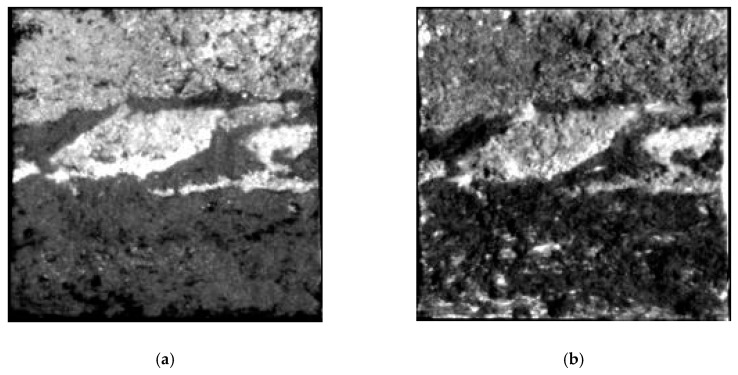
The reflected radiance of scene, (**a**) on flight direction 90° and view zenith −30°; (**b**) on flight direction 90° and view zenith −30°.

The BRF of the mineral powder samples was different from the field targets’ BRF in the previous digital simulation experiment. A physical simulation experiment was conducted to simulate the real imaging process one step further. As shown in Figure 10, a physically simulated scene under the illumination from a physical solar simulator and a physical skylight simulator [37] were imaged from several directions. As the rock sample in the Huangshan cooper mine was not enough for the scene simulation, the Huangshan Dong cooper mine was chosen as the experiment scene. The scaled scene model shown in Figure 11 was developed according to the ASTER GDEM of the scene, the lithological and the mineral distribution map [38], using the rock samples collected from the field. As the BRF changed with the diameter of smashed samples, as shown in Figure 12, the diameter of smashed sample was chosen by comparing the BRF of smashed sample with the BRF of rock sample. The RMSE of BRF simulated is less than 0.01. The illumination onto the scene was simulated with the illumination direction, atmospheric and aerosol modes setting as listed in Table 4. The solar azimuth was simulated by changing the orientation of experiment scene. Finally, the experimental scene was observed at the directions listed in Table 4. Two of the acquired images are shown in Figure 13.

**Figure 14:**
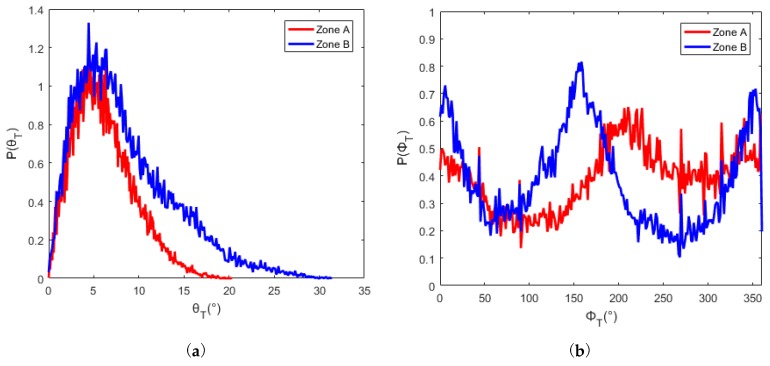
PDFs of slope and aspect in zone A and B: (**a**) the PDFs of slope, (**b**) the PDFs of aspect.

**Figure 15:**
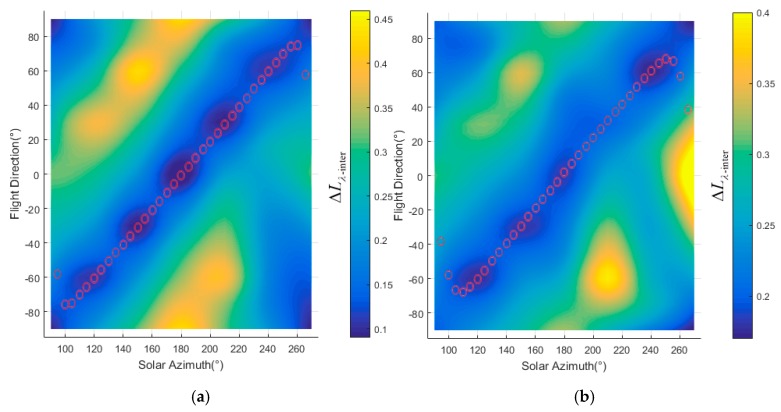
Radiance difference in the overlap area between adjacent stripes: (**a**) the radiance difference in zone A (**b**) the radiance difference in zone B.

The geometric distortion in the image changes with the observation zenith. Hence, in order to calculate the radiance difference $\Delta L_{\lambda -\mathrm{inter}}\left(\phi_{F},\lambda \right)$ between different view zenith with Equation (16), the image observed from two view zeniths must be geometrically corrected with reference to the nadir image. In order to validate the flight direction for different scenes, the two zones whose terrain was rugged and different from each other were defined corresponding to the A and B in Figure 11. The terrain difference between zone A and B are shown as Figure 14. The slope in zone B is slightly greater than that in zone A (the average slope in zone A is 6.2°, the average slope in zone B is 8.5°). The slopes in zone A are generally facing west, and the slopes in zone B are generally facing south or north. The best flight direction for each zone was calculated with Equations (17) and (18), shown as the red circles in Figure 15. The $\Delta L_{\lambda -\mathrm{inter}}\left(\phi_{F},\lambda \right)$ comes to the minimum in the best directions.

## 6. Discussion

**Figure 16:**
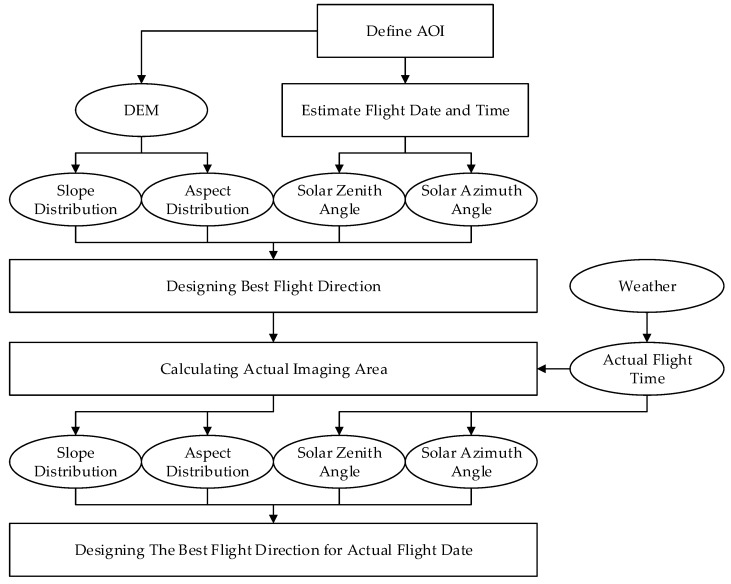
The workflow of the optimized flight direction design method.

The digital and physical simulation experiments showed that $\Delta L_{\lambda -\mathrm{intra}}\left(\phi_{F},\lambda \right)$ and $\Delta L_{\lambda -\mathrm{inter}}\left(\phi_{F},\lambda \right)$ for whickbroom and pushbroom airborne imaging are affected by rugged terrain and could be reduced by adjusting the flight direction. In the case of solo slope, the best flight direction dependent on the illumination direction can be calculated with Equation (19), where $\Delta L_{\lambda -\mathrm{intra}}\left(\phi_{F},\lambda \right)$ and $\Delta L_{\lambda -\mathrm{inter}}\left(\phi_{F},\lambda \right)$ come to the minimum. As to the composite slope scene which is composed of many facets, $\Delta L_{\lambda -\mathrm{intra}}\left(\phi_{F},\lambda \right)$ and $\Delta L_{\lambda -\mathrm{inter}}\left(\phi_{F},\lambda \right)$ of each facet are different. In order to minimize the effect of reflectance anisotropy and terrain in the whole image, the best flight direction is defined as the expectation of the best flight direction for each facet, which would be calculated with Equation (20). By design the flight direction with Equation (20), $\Delta L_{\lambda -\mathrm{inter}}\left(\phi_{F},\lambda \right)$ were reduced from 0.40 to 0.04 in the digital simulation experiment, and were reduced from 0.45 to 0.02 in the physical simulation experiment. Although this radiance non-uniformity can also be corrected by BRDF correction algorithms, the accurate algorithms for rugged terrain scenes are still subjects of ongoing research [22]. It would be effective to reduce the radiance non-uniformity during data acquiring and improve the accuracy of correction by acquiring high performance image at first. A flight direction design method for whickbroom and pushbroom airborne imaging is summarized as in Figure 16. At the beginning of flight planning, the AOI is defined according to the goal of experiment. The solar zenith and azimuth angles are estimated with a software such as SolarBeam [39] and SolarEarthTools [40], according to the flight date and time planed. The flight time window is then designed to avoid the shadow effect and to guarantee sufficient solar irradiance onto the surface, according to the solar zenith and azimuth angles with a flight design software such as Leica Mission Pro [9]. The central time in the time window is chosen to design the best flight direction with Equation (20), accounting for the slope and aspect distribution in the AOI. Before conducting the flight mission, the actual time window is defined considering the weather of flight date. Then the actual imaging area is calculated with the flight design software according to the flight direction designed and time window [7,8,9,11,23]. Then the slope and aspect distribution in the actual imaging area, and the solar zenith and azimuth angles during the imaging process, can be recalculated to adjust the flight direction. Following this workflow, the best flight direction for the actual flight would be determined.

## 7. Conclusions

The anisotropic reflectance of the surface significantly affects the performance of airborne hyperspectral remote sensing image. Current flight direction design methods cannot reduce the effects of rugged terrain as they do for flat terrain. Hence, this paper analyzes the effect of the anisotropic reflectance of the surface to the radiance reached the whickbroom and pushbroom airborne imagers. The radiance gradient in the image and the radiance difference in the overlapping area between adjacent stripes were analyzed for different flight directions and illumination directions. To minimize the radiance gradient and the radiance difference, a method is developed for the composite slope scene illuminated from different directions. Several digital and physical simulation experiments are conducted to demonstrate that the radiance gradient and radiance difference are reduced significantly on the designed flight direction. By cooperating the current flight planning software with the proposed method, a basic workflow of flight direction design is summarized for flight planning. In this workflow, the flight direction is first calculated considering the DEM of AOI and the flight time. Then flight line is planned by the use of current flight planning software with the calculated flight direction. Finally, the flight direction is adjusted before the flight conducted considering the final flight time. With this workflow, the accuracy of airborne imaging can be improved with proper flight planning. As the scene in the physical simulation experiment is flat, the best flight direction in different subzones doesn’t change significantly. More physical experiments will be conducted to simulate the hyperspectral imaging over scenes whose terrains are more rugged in order to analyze the influences of anisotropy of the surface on its retrieved reflectance characteristics.

## Figures and Tables

**Table 1:** Notations used for the description of radiance transfer process.

Symbols	Explanations
$\vec{T}\left(\theta_{T},\phi_{T}\right)$	Normal vector of terrain in horizontal coordinate system
$\vec{I}\left(\theta_{i},\phi_{i}\right)$	Illumination vector (from sun to target) in horizontal coordinate system
$\vec{E}\left(\theta_{e},\phi_{e}\right)$	Observation vector (from observer to target) in horizontal coordinate system
$\theta_{T}$	The slope in horizontal coordinate system
$\phi_{T}$	The aspect in horizontal coordinate system
$\theta_{i}$	The solar zenith angle in horizontal coordinate system
$\phi_{i}$	The solar azimuth angle in horizontal coordinate system
$\theta_{e}$	The observation zenith angle in horizontal coordinate system
$\phi_{e}$	The observation azimuth angle in horizontal coordinate system
$\theta_{i}{}^{\prime }$	The incident zenith angle in local slope coordinate system
$\phi_{i}{}^{\prime }$	The incident azimuth angle in local slope coordinate system
$\theta_{e}{}^{\prime }$	The exit zenith angle in local slope coordinate system
$\phi_{e}{}^{\prime }$	The exit azimuth angle in local slope coordinate system

**Table 2:** Simulation Parameters for solo slope digital simulation experiment.

Attribute	Value
Solar Zenith Angle ($\theta_{i}$)	20°
Solar Azimuth Angle ($\phi_{i}$)	94°–266°
Observation Zenith Angle ($\theta_{e}$)	−30°/0°/+30°
Flight direction ($\phi_{F}$)	90°–270° (5° interval)
Slope	0°–50° (10° interval)
Aspect	0°/180°
Spectral Range	400 nm–2500 nm
Spectral Resolution	10 nm
Atmosphere Model	Mid-Latitude Summer
Aerosol Model	Rural
Visibility	40 km

**Table 3:** Simulation Parameters for composite slope digital simulation experiment.

Attribute	Value
Location	40.0°N, 93.9°E
Solar Direction	Zenith: 17.4°~46.9°, Azimuth: 94.3°~265.8°
Spectral Range	400 nm~2500 nm
Spectral Resolution	10 nm
Atmosphere Model	Mid-Latitude Summer
Aerosol Model	Rural
Visibility	40 km
View Zenith	−30.0°/0°/+30.0°
Flight direction	90.0°~−90.0° (30.0° interval)
Slope	0°~36° (mean: 23°)
Aspect	−180°~180° (mean: 1.4°)

**Table 4:** Simulation Parameters for composite slope physical simulation experiment.

Attribute	Value
Location	40.0°N, 93.9°E
Solar Direction	Zenith: 48°, Azimuth: 90°~270° (30.0° interval)
Atmosphere Model	Mid-Latitude Summer
Aerosol Model	Rural
Visibility	40 km
View Zenith	−30.0°/0°/+30.0°
Flight direction	−90.0°~+90.0°
Slope	0°~50.2° (mean: 8.6°)
Aspect	−180°~180° (mean: 7.5°)
